# Analytical Method Development for Sodium Valproate through Chemical Derivatization

**DOI:** 10.1155/2020/5672183

**Published:** 2020-01-31

**Authors:** Murad Abualhasan, Nihaya Wasif Odeh, Ghadeer Naser Younis, Oyoun Fadel Zeidan

**Affiliations:** Department of Pharmacy, Faculty of Medicine and Health Sciences, An-Najah National University, P.O. Box 7, Nablus, State of Palestine

## Abstract

**Background:**

Sodium valproate has anticonvulsant activity and is structurally different to conventional antiepileptic drugs. The problem with valproic acid is the lack of a chromophore, which means that gas chromatography is the sole assay methodology. The introduction of benzoyl and phenyl groups to the molecule is a useful derivatisation, which enables the creation of detectable chromophores for HPLC analysis for pharmaceutical dosages as well as biological systems. *Methodology*. Sodium valproate was derivatised by the addition of a chromophore to its structure by introducing a methyl benzoyl or a phenyl group. Trichlorophenol and 2-hydroxyacetophenone were used to introduce phenyl and benzoyl groups to valproic acid, respectively. The reaction used was estrification reaction using coupling agents. An analytical method was then developed and validated using reverse-phase HPLC. The method was validated for parameters like linearity, range, accuracy precision, and robustness.

**Results:**

The developed method was easy and feasible and can be applied to both routine analysis and bioanalysis. The method was very sensitive and could quantify valproic acid at a very low concentration of 0.75 × 10^−5^ mg/ml. The developed method was found to be linear (*R*^2^ = 0.997), accurate, precise, and robust.

**Conclusion:**

The proposed chemical derivatisation and the developed analytical method are novel. The developed analytical procedure is the first of its kind; it is easy and feasible and can be used to quantify and detect sodium valproate at very low concentrations compared to other available methods in the literature.

## 1. Introduction

**Figure 1:**
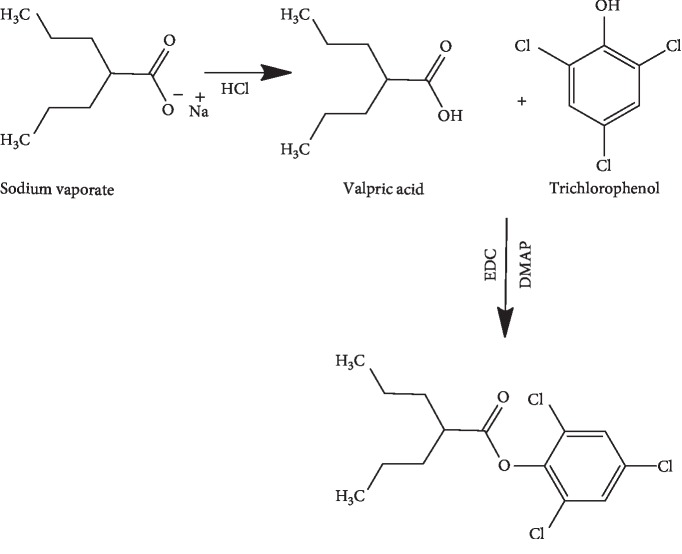
Valproic acid.

Valproic acid (VPA) has a broad spectrum of anticonvulsant activity but is structurally different to conventional antiepileptic drugs (Figure 1). Valproic acid is one of the antiepileptic drugs approved by the U.S. Food and Drug Administration (FDA) for migraine prevention [1–4].

Valproic acid is considered a safer alternative to phenytoin for the treatment of adult onset epilepsy [5–8]. Side effects including anorexia, vomiting, and sleep alterations were the most commonly reported in clinical records [9–14].

Sodium valproate is the sodium salt of valproic acid (VPA). There are several trade names for both materials, including Depalept (sodium valproate) in the form of an enteric coated tablet and Depalept Chrono (mix of both) in the form of a prolonged release tablet [15, 16]. Sodium valproate is rapidly absorbed after oral administration, reaching peak blood levels within 1 to 4 hours [1].

The chemical structure of valproic acid lacks a chromophore, and hence, it has low absorption, which makes it more difficult to detect at low concentrations as it lacks a suitable chromophore. Valproic acid has only weak UV absorbance in the low wavelength range [17, 18].

Chemical derivatisation techniques have been used for classical UV absorption or fluorescence analysis in solution. There are several reasons why chemical derivatisation is useful in liquid chromatography: derivatisation blocks hydrogen bonding sites and reduces the polarity of compounds. Converting alcohols or carboxylic acids to esters greatly improves chromatography. Derivatisation is also used in chromatography for the confirmation of sample identity [19].

During the past decades, multiple analytical methods have been developed to quantify valproic acid in dosage forms, plasma and animal tissues, including high-performance liquid chromatography. However, these methods lack sensitivity and are not capable of analysing the drug at very low concentrations [20–22].

The objective of this study is to derivatise sodium valproate to increase its detection in the UV detection range. An easy and feasible chemical derivatisation method will be capable of changing valproic acid or its sodium salt to be detected at very low concentrations. The developed method can be adapted in the routine analysis of valproic acid in pharmaceutical dosages as well as biological systems. The method will be validated for parameters like linearity, range, accuracy precision, and robustness.

The proposed chemical derivatisation and the developed analytical method are novel. To the best of our knowledge, this procedure is the first of its kind. The developed method is intended to be used in the quantification and detection of sodium valproate at very low concentrations compared to other available methods in the literature.

## 2. Methodology

### 2.1. Chemical

Different reagents were used throughout the research project. All the reagents used were of analytical grade and were purchased from reliable resources. Sodium valproate was given as a gift from Birzeit Palestine pharmaceutical company (1-ethyl-3-(3-dimethylaminopropyl) carbodiimide hydrochloride (EDC) and 4-dimethylaminopyridine (DMAP)) were purchased from Sigma, Germany. HPLC-grade acetonitrile and methanol were purchased from Sigma UK.

2,4,6-Trichlorophenol and 2-hydroxyacetophenone were purchased from Sigma USA. The Micropore Water System was used to generate HPLC-grade water.

### 2.2. Instrumentation

Different instruments were used in the project, including a magnetic stirrer hotplate (LabTech/LMS-1003), a weighing balance (Adventurer-OHAVS-AR2140), a Rota Evaporator (Heidolph-Rova-100), an oven (UNB400–C407.0109), a sonicator (MRC/DC-200H), a UV spectrophotometer (Jenway/7315), ATR-FTIR (Thermo Fisher Nicolet IS5), a shaker (MEMMERT, GMBH), an HPLC Chromatograph (BREEZE 1525, WATERS), and a micropore system (Veolia Water PF3, UK).

### 2.3. Chemical Derivatisation

**Scheme 1:**
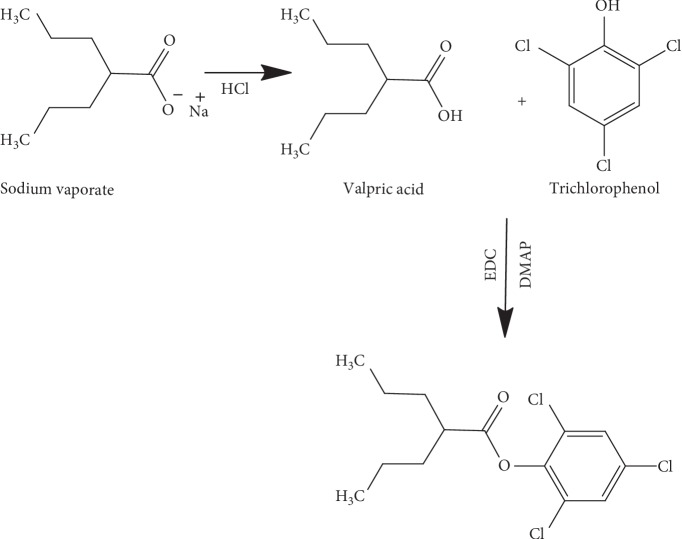
Reaction of valproic acid with trichlorophenol.

**Scheme 2:**
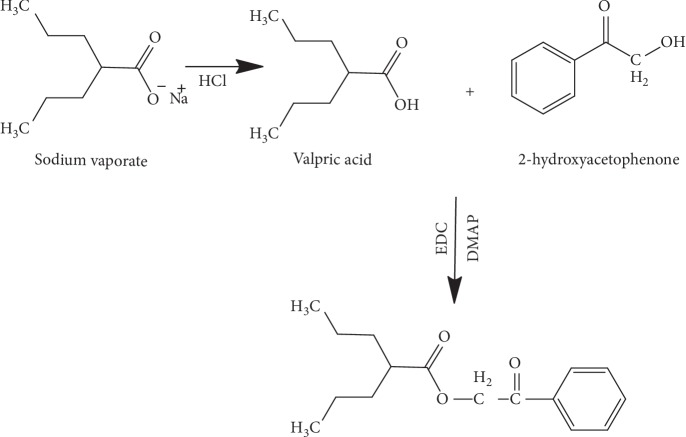
Reaction of valproic acid with 2-hydroxyacetophenone.

Sodium valproate was derivatised by adding a chromophore to its structure by introducing a methyl benzoyl or phenyl group. The reaction followed the chemical reaction summarised in Schemes 1 and 2.

#### 2.3.1. Preparation of Valproic Acid from Na Valproate

Sodium valproate (Na-valproate) was converted to valproic acid by weighing Na valproate (0.5 g) and dissolving it in 25 ml water. To this solution, 1M HCl (4 ml) was added, and it was stirred using a magnetic bar for 30 min. The reaction was extracted with dichloromethane, and the organic layer was taken and dried under a vacuum using the Rota Evaporator.

#### 2.3.2. Preparation of Standard Valproic Acid Derivative of Trichlorophenol and 2-Hydroxyacetophenone

The valproic acid produced was used for further derivatisation. A mixture of 1 mmol (145 mg) of valproic acid, 1 mmol (200 mg) of EDC, 1 mmol (122 mg) of DMAP, and 1 mmol (197 mg) of trichlorophenol. The reaction was placed under vacuum argon. Dichloromethane (DCM) 8 ml was added to it, and the mixture was allowed to stir for 24 hr. The reaction was monitored using thin layer chromatography (TLC) with a mobile phase (7 : 1) of DCM : methanol. The product was purified by column chromatography using (7 : 3) hexane : ethylacetate.

The same procedure was followed to derivatise valproic acid with 2-hydroxyacetophenone in which 27.58 mg (1 mmol) of it was added to the reaction.

#### 2.3.3. Derivatisation of Sodium Valproate for HPLC Analysis Using Trichlorophenol

Na valproate 33.5 mg (0.2 mmol) was dissolved in 1 ml acetonitrile. To this, 200 *μ*l of HCl was added and allowed to stir for 30 minutes. DMAP solution (30 mg/0.5 ml acetonitrile) and EDC (40 mg/0.5 ml acetonitrile) were added to the Na valproate solution and were mixed together. Trichlorophenol (40 mg/0.5 ml acetonitrile) was then added to the mixture and stirred for 2 hours. The mixture was then dried under nitrogen. The dried powder was diluted to 10 ml with acetonitrile, which was then ready to be injected into the HPLC.

#### 2.3.4. Derivatisation of Sodium Valproate for HPLC Analysis Using 2-Hydroxyacetophenone

The same procedure followed for trichlorophenol was used for 2-hydroxyacetophenone. The procedure was performed using three levels of Na valproate. Na valproate (10 mg, 20 mg and 30 mg) was weighed, and each amount was dissolved in 1 ml acetonitrile in a separate tube. 2-Hydroxyacetophenone (9.45, 18.8 and 28.32 mg) was then added to it. To each tube, 30 mg DMAP and 40 mg EDC were added. Each mixture was allowed to stir for 1 hour and then dried under nitrogen. The dried powder was diluted to 10 ml with acetonitrile and was then ready to be injected into the HPLC.

### 2.4. HPLC Method Development

The HPLC method development was performed using the 2-hydroxyacetophenol reaction. This particular reaction was chosen to be the adapted analytical method due to its better separation. Moreover, this reagent introduces benzoyl to the valproic acid structure, which has an extended conjugation, so it will shift the absorbance of the parent drug to a more hyperchromic and bathochromic shift, while the reaction using trichlorophenol introduced a phenol group only.

The reaction mixture was injected into the HPLC in different compositions of the mobile phase using methanol and acetonitril mixed with water in different percentage, and the percentage of organic solvent ranged from 50–90% (v/v). The detection wavelength was examined in the range of 230–254 nm. Moreover, the mobile phase was run at a flow rate in the range from 1–2 ml/min). The HPLC condition was set when the best peak shape and retention time were obtained.

### 2.5. HPLC Method Validation

The method was validated for parameters like linearity, range, accuracy, precision, limit of detection (LOD), limit of quantification (LOQ), and robustness/ruggedness.

To evaluate the linearity and range of the method, 5 different test concentrations were prepared: 0.75 mg/1 ml, 1 mg/1 ml, 1.5 mg/1 ml, 2 mg/1 ml, and 3 mg/1 ml. Three separate injections were analysed under the same conditions, and the average reading was recorded. The obtained peak area was plotted against the concentration; the *R*^2^ and regression line equation was recorded.

The accuracy was assessed by adding 1 mg/1 ml valproic acid in addition to some widely used excipients that are usually added in oral dosage forms. The added excipients include starch, magnesium stearate, and carboxymethyl cellulose. The percentage recovery of the test was calculated.

Repeatability precision was established for 3 concentrations around the test concentration (0.7 mg/1 ml, 1.5 mg/1 ml, and 3 mg/1 ml). Three replicates of each concentration were prepared, and the relative standard deviation (RSD) of the result was calculated.

The sensitivity of the method was established by measuring the LOD and LOQ. The LOD is expressed as a concentration that gives a signal-to-noise ratio of approximately 3 : 1, while the LOQ in a sample can be determined with acceptable precision and accuracy with a signal-to-noise ratio of approximately 10 : 1.

The ruggedness/robustness of the method was determined by performing the same trial using small variations in different parameters, including: mobile phase pH, detection wavelength, and flow rate. The conditions of different parameters tested included the following: Mobile-phase composition, UV absorption (*λ*), flow rate, and measurement in different days [23].

## 3. Results and Discussion

### 3.1. Chemical Derivatisation

The chemical derivatisation was successful completed using the coupling regents DMAP and DCC. The TLC results showed a spot that was clearly visualised under UV light. The spot was the reactant used in the derivatisation reaction to introduce conjugation to valproic acid by either trichlorophenol or 2-hydroxyacetophenol. The spot was a dark black with an *R*_f_ value of approximately 0.6. The spot was clearly seen under the UV light compared with valproic acid, which was not seen under UV because it lacks conjugation. The *R*_f_ of the synthesised compound was higher than all the reacting regents due to its increased lipophilicity.

The IR data of the derivatised compound showed a successful esterification reaction of the valproic acid.

### 3.2. Method Development and Validation

The developed methods for HPLC separation were run using reverse-phase chromatography. The HPLC conditions are illustrated in Table 1.

Linearity and range was performed on a sodium valproate in a concentration range of 0.75–0.30 mg/ml. The concentration was plotted against the peak area under the curve (AUC). The calibration curve was linear with *R*^2^ = 0.997, and the regression line equation was

**Figure 2:**
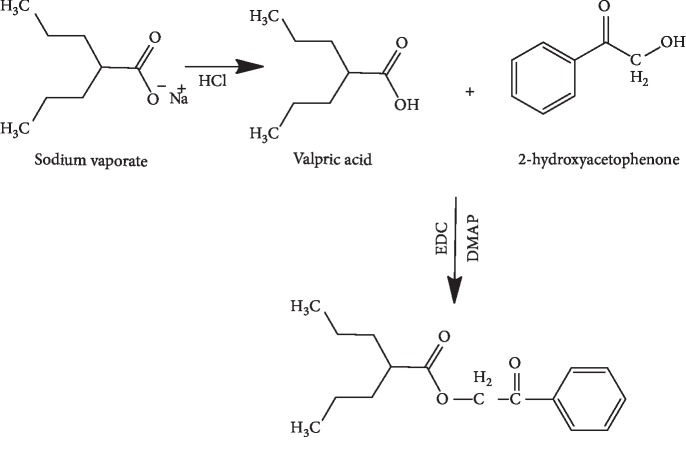
The calibration curve of linearity and range.

 
*y* = 8*E*^+06^*x* + 2*E*^+06^ (Figure 2 and Table 2).

The accuracy of the method was performed by adding commonly used excipients, which include starch (10 mg), carboxymethyl cellulose (10 mg), and Mg stearate (1 mg) in the tested reaction. The resulted peak area of this mixture and the peak area of the standard were used to calculate the percentage assay. The result of the % assay was found to be 99.91 (Table 3).

**Figure 3:**
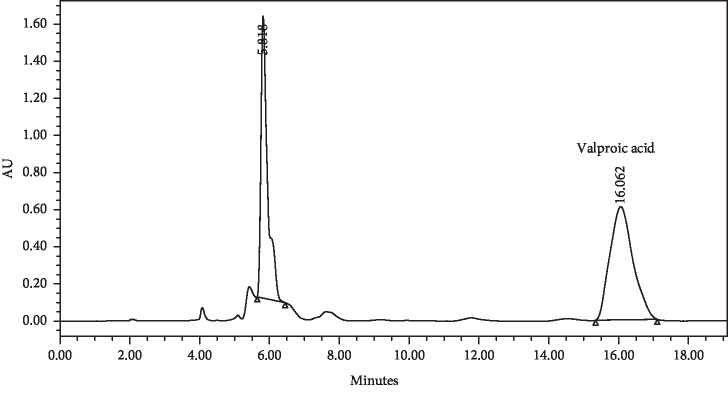
Chromatogram of the derivatised valproic acid.

The generated peak of the derivatised valproic acid was highly separated with high resolution from other interfering peaks, which elute early due to their high polarity (Figure 3).

The results of repeatability and precision showed that the developed method was repeatable (intermediate precision) over the tested range of valproic acid concentrations. The RSD was in the range from 0.3–2.1 (Table 4).

The method was found to be robust under the tested variations mentioned in the methodology section; the injected concentration of sodium valproate was 1 mg/1 ml. The results showed no variability among the generated peaks at the above-mentioned conditions. The RSD of the AUC was found to be 0.807 (Table 5).

The LOD and LOQ of the method provide an indication of the method sensitivity. The minimum quantity of sodium valproate in which the method can detect is expressed as an LOD and was determined by injection-diluted samples of the derivatised valproic acid. The noise : peak ratio of 3 : 1, which represents the LOD, was found to be 0.75 × 10^−6^, while the noise : peak ratio of 10 : 1 was determined as the LOQ and was found to be 0.75 × 10^−5^.

Finally, the method was found to be selective for the generated peak of the derivatised valproic acid. The peak was well separated from the other eluting peaks, which have a shorter retention time due to its hydrophilicity compared to the more lipophilic derivatised valproic acid. Moreover, the derivatised peak was symmetrical with an acceptable theoretical plate (Figure 3).

**Figure 4:**
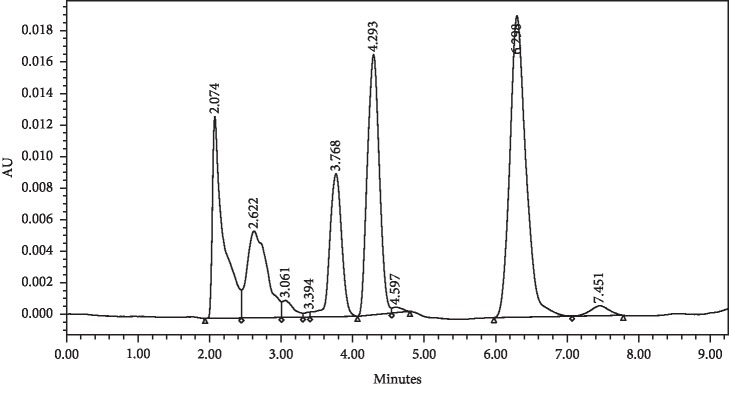
The HPLC chromatogram of underivatised Na valproate.

As expected, the developed method increased the absorption of the valproic acid when derivatised compared with the underivatised valproic acid. We can clearly notice the huge difference in absorbance between the derivatised and underivatised valproic acid. The absorbance of underivatised valproic acid at concentration of 1 mg/ml showed an absorbance less than 0.02 (Figure 4).

The method is simple and feasible and can be applied to both routine analysis as well as bioanalysis, and it is also capable of measuring very small quantities of valproic acid in biological systems.

## 4. Conclusion

In this research project, we could successfully develop an analytical method for Na valproate through the introduction of conjugation. The conjugation was introduced by reacting valproic acid with 2-hydroxyacetophenone to form an ester with the drug. The introduced benzoyl ring made the drug more lipophilic. The eluted peak showed sufficient absorbance to be quantified at much lower concentrations compared to the parent drug. The developed method is easy and feasible and can be applied to both routine analyses as well as bioanalysis.

## Figures and Tables

**Table 1:** HPLC conditions.

Flow rate	1 ml/1 min
Wavelength (*λ*)	254 nm
Mobile phase	(ACN : WATER; 80 : 20)
Stationary phase	C18; 4.6 ∗ 250 mm
Column temperature	20°C
Injection volume	20 *μ*L
Run time	19 min

**Table 2:** The linearity and range.

Conc. (mg/ml)	AUC
0.75	7278144
1.0	10227279
1.5	13874096
2.0	17437422
3.0	25213906

**Table 3:** The assay accuracy.

	AUC	Average	% assay
Sample	11138767	11197932	99.91835
	11257096		
Standard	11157087	11207082	
	11257077		

**Table 4:** Repeatability results of three different concentrations of valproic acid.

Conc. mg/1 ml	AUC	Average	% RSD
0.75	7106403	7278144	2.155926
	7314015		
	7414014		

1.5	13833360	13874096	0.376175
	13932926		
	13856002		

3.0	25007484	25213906	0.731791
	25362807		
	25271427		

**Table 5:** The robustness results for different variable parameters of the HPLC.

Condition	AUC	% RSD
Normal condition	11057086	0.8076379
	11257075	
*λ* = 248 nm	11056087	
	11057097	
*λ* = 252 nm	11157087	
	11257097	
Flow 1.1	11059085	
	11257073	
Mobile 25/75	11157089	
	11257976	
1st day	11157084	
2nd day	11057043	
Average	**11148907**	

## Data Availability

The data used to support the findings of this study are included within the article.
